# Seroprevalence of *Toxoplasma gondii* among HIV Positive Patients under Surveillance in Greek Infectious Disease Units: A Screening Study with Comparative Evaluation of Serological Methods

**DOI:** 10.3390/pathogens13050375

**Published:** 2024-04-30

**Authors:** Chrysa Voyiatzaki, Apollon Dareios Zare Chormizi, Maria E. Tsoumani, Antonia Efstathiou, Konstantinos Konstantinidis, Dimitrios Chaniotis, Georgios Chrysos, Aikaterini Argyraki, Vasileios Papastamopoulos, Marika Kotsianopoulou

**Affiliations:** 1 Department of Biomedical Sciences, Division of Medical Laboratories Science, University of West Attica, 12243 Athens, Greece; 2 Immunology of Infection Group, Department of Microbiology, Hellenic Pasteur Institute, 11521 Athens, Greece; 3 Department of Medicine, Laboratory of Biology, Democritus University of Thrace, Dragana, 68100 Alexandroupolis, Greece; 4 Second Department of Internal Medicine, Tzaneio General Hospital of Piraeus, 18536 Athens, Greece; 5 Department of Internal Medicine, Sotiria Thoracic Diseases General Hospital, 11527 Athens, Greece; 6 5th Department of Internal Medicine, Infectious Diseases Unit, Evaggelismos General Hospital, 10676 Athens, Greece; 7 Department of Public Health Policy, School of Public Health, University of West Attica, 11521 Athens, Greece

**Keywords:** opportunistic infection, infectious disease, toxoplasmosis, Europe/Greece, comparative serology, ELISA, IFAT, WB

## Abstract

This study aims to screen for IgG antibodies against *Toxoplasma gondii* (*T. gondii*) in the sera of 155 newly diagnosed Human Immunodeficiency Virus (HIV) positive patients under surveillance in Greek Infectious Disease Units. Additionally, risk factors based on patient demographics were examined, and a comparative evaluation of commercially available serological methods was conducted. Three methods were employed to detect IgG antibodies against *T. gondii*: Enzyme-Linked Immunosorbent Assay (ELISA), Indirect Immunofluorescence Antibody Test (IFAT), and Western Blot (WB), which was used as a reference here. Forty-nine sera samples were true-positive for IgG antibodies against *T. gondii*, resulting in a 31.61% positivity rate, and the immunoassay test statistical reliability analysis resulted in higher IFAT accuracy (90.97%) compared to ELISA (76.26%). Furthermore, statistical analysis of demographic and immunological data included in the study placed female and foreign/non-Greek individuals at 2.24 (*p* = 0.0009) and 2.34 (*p* = 0.0006) times higher risk of positive *T. gondii* IgG testing compared to their male and Greek counterparts, respectively. Our findings on positivity rates and comparative serology underscore the importance of early and suitable screening measures for newly diagnosed HIV+ patients to mitigate the life-threatening outcomes that may arise from a potential subsequent *T. gondii* activation.

## 1. Introduction

The *Toxoplasma gondii (T. gondii)* is a protozoan intracellular Apicomplexan parasite that infects warm-blooded animals, including humans. It has a complex morphology and can take on different forms depending on the stage of its life cycle. Three forms of *T. gondii* can infect humans: rapidly replicating tachyzoites during the acute phase of infection, slowly replicating bradyzoites within tissue cysts during the chronic phase, and sporozoites within released oocysts into the environment. The parasite can be transmitted to humans by consuming contaminated and undercooked meat with bradyzoite cysts, contaminated soil or water, and unwashed fruits and vegetables that contain oocysts, from animal to human and from mother to child during pregnancy. In rare cases, through organ transplants and blood transfusions [1]. Furthermore, the detection of *T. gondii* tissue cysts in human semen raises the possibility of sexual transmission of the parasite [2]. In Europe, it was reported that contact with contaminated soil was responsible for 6–17% of infections, while the consumption of contaminated meat was attributed to 30–63% of infections [3]. 

It is estimated that 30% of the world’s population is infected with *T. gondii*. Among Human Immunodeficiency Virus positive (HIV+) patients, the global pooled prevalence of *T. gondii* infection is as high as 35.8%, with the most significant burden observed in low-income countries. However, there exists a dearth of country-level studies on the prevalence of *T. gondii* infection among HIV+ patients, resulting in considerable variation observed within and between countries, thus potentially leading to an underestimation of the true prevalence [4]. Strains I, II, and III are the most commonly found in Europe, and they exhibit variability in terms of their virulence, growth rate, and cysts-forming ability [5]. Type II strains are responsible for most human infections [6]. Noteworthy, the immunity against one strain may not necessarily confer protection against another [3]. 

In immunocompetent individuals, latent *T. gondii* infection is considered asymptomatic. However, in the case of immunosuppression, the infection can become active, particularly in those with HIV, leading to cerebral toxoplasmosis, which is the most common opportunistic central nervous system disease in such patients and exhibits symptoms such as headache, cognitive deficits, fever, limb paresis, speech disorders, coordination, and seizures [5,7]. The risk of developing cerebral toxoplasmosis appears consistent between the pre-Antiretroviral Therapy (pre-ART) and ART eras; however, its incidence and mortality have decreased [7]. A systematic review and meta-analysis reported that despite using ART, cerebral toxoplasmosis accounted for 6% of hospital admissions among HIV+ patients [8]. 

Regarding laboratory evaluation, while Polymerase Chain Reaction (PCR) is efficacious in detecting *T. gondii* DNA, it does not provide information about the immune status of patients or the potential for chronic infection. Therefore, employing serological tools in tandem with PCR is recommended, as they offer a cost-effective way to acquire supplementary and invaluable data. A variety of serological tests are commercially available for the detection of IgM and IgG antibodies against *T. gondii*. Nonetheless, their accuracy is variable, as manufacturers employ different antigens, leading to discordance between techniques [9]. Manufacturers usually do not mention which antigens are used, making it difficult to compare the methods [10]. Initially, antibodies target surface antigens; therefore, serological methods based on membrane antigens may be more efficient in detecting IgG earlier than methods that employ whole parasites [3].

Following the initial increase of IgM, IgG can be detected within 1 to 3 weeks and reaches a plateau after 2–3 months. Eventually, IgG levels decrease, but the remaining titles are detected for life [3]. In primary infection, the appearance of IgM, the increase of IgG, and the subsequent stabilisation of IgG with possible persistence of IgM are determined. In activation, the increase in IgG titers and their subsequent stabilisation is determined [9]. However, IgM cannot reliably indicate a recent infection as it may still be detected after two years following the initial infection. Therefore, IgG avidity is determined, i.e., the measurement of the binding power of the antibody, which is based on the progressive increase in its affinity for the target antigen [3]. 

Enzyme-linked Immunosorbent Assay (ELISA) is commonly used in most laboratories, although the accuracy of commercially available assays can vary significantly [3]. Often, antibody concentrations are found near the detection limit, and manufacturers establish a grey zone corresponding to low antibody titers, which can result in uncertain or equivocal results [11]. The Sabin–Feldman or Dye Test (DT) is the gold standard in the serodiagnosis of toxoplasmosis. It is based on the lysis of parasites in the presence of complement [3]. However, it is complex to perform and standardise since it requires the maintenance of live tachyzoites and is applied in a few laboratories [12]. A good alternative, the test LDBIO TOXO II Western Blot IgG kit (Lyon, France), has been proposed as a confirmatory technique since its results appear to be consistent with those of DT, providing 100% specificity and 99.2% sensitivity, and has been used as a reference technique in cases of discordant, equivocal, or borderline results, preventing unnecessary and expensive serological follow-up [10,11,12]. The LDBIO TOXO II Western Blot IgG diagnostic test strips are coated with natural *T. gondii* antigens. 

Following an HIV diagnosis, patients must undergo an *T. gondii* IgM and IgG test. In the event of a positive result, anti-toxoplasmosis prophylaxis should be administered alongside Highly Active Antiretroviral Therapy (HAART). On the other hand, in the event of a negative result, patients are informed on how to protect against a primary infection [9]. The administration of anti-*Toxoplasma* treatment is warranted for patients with suspected active toxoplasmosis who have previously tested positive for IgG antibodies. Moreover, for patients with a CD4+ count < 200 cells/μL and a positive PCR detection of *T. gondii* DNA, elevated serum IgG levels can indicate a higher risk of activation and subsequent development of rapidly progressive and potentially fatal cerebral toxoplasmosis. Therefore, early and accurate diagnosis is critical, although the delay between the rise in antibody titers and the occurrence of cerebral toxoplasmosis is not well-defined [3,7,8,13].

Limited information exists in Greece about the seroprevalence of *T. gondii* among HIV+ patients. Here, we conducted a country-level study to investigate the prevalence of *T. gondii* IgG antibodies in HIV+ patients. For this purpose, we screened 155 HIV+ samples with Enzyme-Linked Immunosorbent Assay (ELISA), Indirect Immunofluorescence Antibody Test (IFAT), and Western Blot (WB). WB was used as a confirmatory technique to resolve discrepancies and perform a comparative evaluation of the serological methods.

## 2. Materials and Methods

### 2.1. Study Site

The experimental research was conducted at the National AIDS Reference Center of Southern Greece and the Laboratory of Molecular Microbiology and Immunology, Department of Biomedical Sciences, University of West Attica, Athens, Greece.

### 2.2. Sera Samples

The sera samples utilised in this study were provided by the National AIDS Reference Center of Southern Greece in 2020. The samples were stored at −20 °C, and unnecessary thawing and freezing cycles were prevented.

### 2.3. Laboratory Techniques

All methods were performed according to the manufacturers’ instructions. Before conducting any experiments, all reagents and sera to be tested were allowed to room temperature.

#### 2.3.1. ELISA

To detect IgG antibodies against *T. gondii* using the ELISA method, we employed the *Toxoplasma Gondii* IgG ELISA kit from DRG Instruments GmbH in Marburg, Germany, which utilizes microtiter wells coated with inactivated *T. gondii* soluble antigen as a solid phase. The serum samples were diluted to 1 + 100 with sample diluent, and anti-IgG conjugate was utilized. The positive results were detected using incubation with Tetramethylbenzidine (TMB) substrate, and the reaction was subsequently halted by adding sulfuric acid. The Optical Density (OD) was measured at a test wavelength of 450 nm, while a reference filter was set to 620 nm to rectify any non-specific absorbance that might have occurred, as recommended by the manufacturer. The validity of the method was determined based on the OD values for the negative control (less than 0.20), standard 1 (between 0.35 and 0.85), standard 2 (between 0.75 and 1.50), standard 3 (between 1.00 and 2.00), and positive control (between 0.65 and 3.00).

#### 2.3.2. IFAT

The MASTAFLUORTM *Toxoplasma* IgG kit (Mast Group, United Kingdom) was used to detect IgG antibodies against *T. gondii* using the IFAT method. All wells in the kit were coated with purified antigens of *T. gondii*. The serum samples were diluted to a ratio of 1:64 with diluent. FITC-conjugated IgG was used, and the presence of a homogenous green fluorescence of the cytoplasmic membrane of *T. gondii* tachyzoites determined a positive result. The cytoplasm might show green staining or even fluorescence together with the cytoplasmic membrane.

#### 2.3.3. WB

The LDBIO TOXO II Western Blot IgG kit (LDBIO Diagnostics, Lyon, France) was used to detect IgG antibodies against *T. gondii* by the WB method and to confirm the results of the previous tests. *T. gondii* antigens were separated using electrophoresis and bound using electroblotting to nitrocellulose membranes. Alkaline phosphate-anti-human IgG conjugate was used, and specific bands were formed by adding NBT/BCIP substrate. A positive result is defined by the formation of at least three bands at 30 kDa, 31 kDa, 33 kDa, 40 kDa, and 45 kDa, including the 30 kDa band.

### 2.4. Data Analysis

The reliability of the examined ELISA and IFAT immunoassay tests was assessed by comparing the qualitative outcomes (positive or negative only, excluding doubtful ELISA grey zones) of T. gondii IgG detection for each immunoassay against those of LDBIO TOXO II Western Blot method, which was used as a reference in this study. Statistical evaluation of variations in sensitivity, specificity, Positive Predictive Value (PPV), Negative Predictive Value (NPV), precision, and accuracy among the examined immunoassay tests was conducted using the χ^2^ test, with a significance level of 95%, and the Wilson–Brown method to compute confidence intervals. Similarly, statistical analysis of the various demographic and immunological data sourced from the HIV+ patients was also conducted using the χ^2^ test, with a significance level of 95%, and the Wilson–Brown method to compute confidence intervals.

## 3. Results

### 3.1. Demographics of HIV+ Patients

The study sample was composed of 155 patients with an average age of 41, with 111 (71.61%) of the participants identifying as male and 42 (27.1%) as female, while 2 (1.29%) were unclassified. The sample also included 90 (58.06%) Greek nationals and 62 (40%) foreign nationals. Regarding HIV testing, 111 (71.61%) of the participants were tested for diagnostic purposes, 21 (13.55%) were intravenous drug users, and 20 (12.9%) underwent testing as a preventive measure.

### 3.2. Laboratory Diagnosis

**Figure 1:**
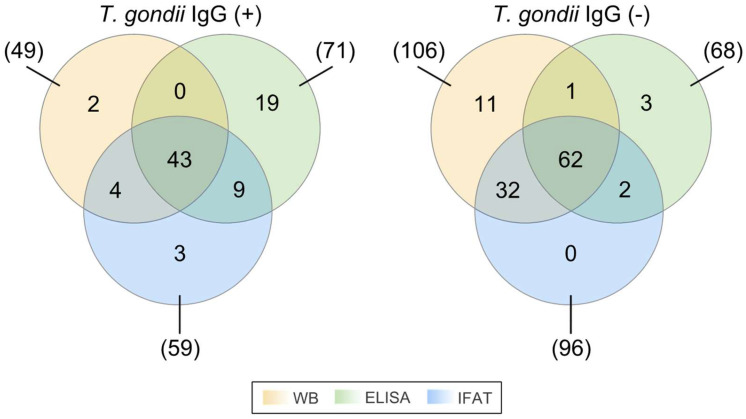
Symmetric three-set Venn diagram of the *Toxoplasma gondii* (*T. gondii*) IgG detection results output by the three examined assays, namely Western blot (WB), Enzyme-linked immunosorbent assay (ELISA), and Indirect Immunofluorescence Antibody Test (IFAT). All possible intersections and exclusive regions of each different set of positive [*T. gondii* IgG (+)] or negative [*T. gondii* IgG (-)] assay results have been drawn and coloured accordingly. The displayed numbers between the parentheses represent the total number of positive or negative results of the respective assay designated by a line, while the numbers within the colourful regions correspond to the number of positive or negative assay results which are unique to a specific assay or union of assays.

Out of 155 samples from HIV+ patients, 71 (45.81%, C.I. 95% 38.16–53.66%) tested positive for IgG antibodies against T. gondii with the ELISA method, excluding doubtful ELISA grey zones, while 59 (38.06%, C.I. 95% 30.8–45.91%) were positive with the IFAT method (Figure 1). Confirming positive results from either ELISA or IFAT or both with the WB method, 47 (30.32%, C.I. 95% 23.6–37.96%) samples were identified as positive, while 43 (27.74%, C.I. 95% 21.3–35.26%) tested positive with all three methods. The WB method also detected 2 (1.29%, C.I. 95% 0.35–4.58%) positive samples that were negative with the other two methods, resulting in 49 total positives out of the 155 samples (31.61%, C.I. 95% 24.81–39.3%). Notably, 31 (20%, C.I. 95% 14.46–26.99%) samples were determined to be true false-positives, as they were negative with WB despite being positive with at least one other test. Of these, 19 (12.26%, C.I. 95% 7.99–18.35%) were false positives with ELISA, 3 (1.94%, C.I. 95% 0.66–5.54%) were false positives with IFAT, and 9 (5.81%, C.I. 95% 3.09–10.67%) were falsely identified as positive with both ELISA and IFAT tests for the same sample. Additionally, 16 (10.32%, C.I. 95% 6.45–16.11%) samples produced doubtful results (grey zones) with ELISA and were excluded from the statistical analysis regarding method reliability (Supplementary File S1).

### 3.3. Statistical Analysis

The reliability of the examined immunoassay tests was determined in terms of relative sensitivity, specificity, PPV, NPV, precision, and accuracy against LDBIO TOXO II Western Blot, which was used as the reference method in this study. The yielded results after the method reliability statistical analysis indicated considerably higher sensitivity, specificity, PPV, NPV, precision, and accuracy for IFAT compared to ELISA, returning 95.92% (C.I. 95% 86.29–99.27%), 88.68% (C.I. 95% 81.25–93.40%), 79.66% (C.I. 95% 67.73–87.96%), 97.92% (C.I. 95% 92.72–99.63%), 79.66% (C.I. 95% 67.73–87.96%), and 90.97% (C.I. 95% 85.41–94.54%) for these metrics, respectively (Table 1).

In addition, statistical analysis of the included demographic and immunological data was performed and revealed statistically significant relationships between *T. gondii* IgG presence and both gender and nationality of the examined HIV+ patients (Table 2). More specifically, female HIV+ patients in Greece are more prone to positive *T. gondii* IgG testing than male HIV+ patients (2.24 times higher risk, *p* = 0.0009). Similarly, foreign/non-Greek HIV+ patients are also more likely to be detected with *T. gondii* IgG compared to Greek HIV+ patients (2.34 times higher risk, *p* = 0.0006). No other statistically significant relationships were detected.

## 4. Discussion

Limited information exists on the incidence of *T. gondii* infection among HIV+ patients in Europe [14,15], and studies on its seroprevalence are scarce. HIV+ patients are at risk of developing opportunistic infections such as toxoplasmosis, Pneumocystis jirovecii pneumonia, and leishmaniasis due to their compromised immune system. While cerebral toxoplasmosis, typically presenting with one or more ring-enhancing brain lesions, is the most common manifestation in these patients, a meta-analysis revealed a high incidence and severity of ocular complications in these individuals. Therefore, it is of paramount importance to thoroughly comprehend the prevalence of toxoplasmosis among HIV+ patients, as this knowledge forms the basis for developing effective management and prevention strategies [16]. 

Serology still plays a crucial role in diagnosing, preventing, managing, and surveying toxoplasmosis. IgG antibodies persist lifelong at residual titers. Therefore, a positive test result is the only way to identify immunocompromised patients at risk of clinical reactivation. This reactivation is characterised by an increase in IgG titers followed by stabilisation. However, there is a high degree of individual variation in the quantity of circulating IgG [9]. Additionally, seroprevalence studies are of paramount importance to avoid underreporting, given the considerable variation in *T. gondii* IgG prevalence rates across different countries [4]. Also, the implementation of comparative evaluation studies can play a crucial role in enhancing the precision of laboratory testing, ultimately leading to improved accuracy of experimental results and timely delivery of appropriate care to patients [17]. 

The LDBIO TOXO II Western Blot test is particularly useful in cases where *T. gondii* IgG titers must be reliably determined. It has a remarkable capability of distinguishing between low-positive and negative sera with a specificity of 100% and sensitivity of 99.2% compared to the DT. Due to this fact, the LDBIO TOXO II Western Blot was used as the reference method in this study to assess the reliability of the examined ELISA and IFAT immunoassays in terms of sensitivity, specificity, PPV, NPV, precision, and accuracy. After the comparative analysis of the qualitative immunoassay test results (positive or negative only, excluding doubtful ELISA grey zones), IFAT returned the highest rates of sensitivity, specificity, PPV, NPV, precision, and accuracy, yielding the least possible false-positive and false-negative results (Figure 1, Table 1). Two additional samples were found to be true-positives with the LDBIO TOXO II WB method, used as a reference here, but false-negatives with both the ELISA and IFAT methods. Multiple negative serological tests do not guarantee true IgG negativity of the sera due to inadequate sensitivity, as observed in other studies [10]. Moreover, 31 samples were false positives with ELISA and/or IFAT. The ELISA assay was performed individually since the high-reliability diagnostic kit does not necessitate the performance of replicates for assay reliability.

A recent study investigated false-positive Architect Toxo IgG results and discovered that these results may be linked to reactivity against a cytoplasmic protein of 35kDa called GRA8, which is present in the tachyzoite and bradyzoite stages of the parasite. This may be due to past exposure to parasites genetically closely related to *T. gondii*, such as *Hammondia hammondi* and *Neospora caninum*, which are not known to cause human infection [10]. In their study, to confirm the presence of specific *T. gondii* IgG, the LDBIO TOXO II Western Blot IgG kit was also used.

Regarding the antigens used in our study, according to the manufacturers’ instructions, (i) the ELISA kit contains inactivated *T. gondii* soluble antigen (strain RH) showing no cross-reactivity for *Entamoeba histolytica*, *Giardia lamblia*, *Schistosoma*, *Toxocara*, *Strongyloides*, and *Echinococcus*, (ii) the IFAT kit incorporates purified antigens of *T. gondii*, and (iii) the WB kit uses antigens of *T. gondii* with no prior information. However, the presence on the strip of a minimum of 3 bands out of specific bands 30, 31, 33, 40 and 45 and the inclusion of the band at 30 kDa declares a result as positive, and in the literature, the membrane protein of 30 kDa called Surface Antigen 1 (SAG1) is only found in the tachyzoite stage of the parasite. 

Apart from the comparative reliability analysis of the studied immunoassays, the demographic and immunological data of the 155 examined HIV+ patients were also analysed, revealing statistically significant relationships between *T. gondii* IgG presence and both gender and nationality (Table 2). According to the results, HIV+ female individuals in Greece exhibit a higher likelihood of testing positive for *T. gondii* IgG compared to their male counterparts, with a risk of approximately 2.24 times higher (*p* = 0.0009). Furthermore, foreign or non-Greek HIV+ patients are more prone to *T. gondii* IgG detection compared to Greek HIV+ patients, showing a risk of approximately 2.34 times higher (*p* = 0.0006). On the contrary, no statistically significant relationship emerged between *T. gondii* detection and HIV+ patients with former intravenous drug use. 

Our positivity rate of 31.61% is consistent with the estimated global prevalence of *T. gondii* at 30%. Higher prevalence rates are typically observed among HIV+ patients; however, our finding could be justified by the fact that we tested newly diagnosed HIV+ patients. Considering the risk factors, hormonal influences on the immune response to *T. gondii* may vary by gender, explaining the higher seroprevalence rates found in females compared to males in our study. Specifically, sex hormones such as oestrogen and progesterone may increase the infective capacity of *T. gondii* and consequently affect the progression of toxoplasmosis. Evidence suggests that *T. gondii* infection induces changes in the expression of hormonal receptors [18]. Furthermore, the higher prevalence in foreign or non-Greek HIV+ patients may be attributed to environmental exposure prior to their arrival in Greece. *T. gondii* is more commonly observed in regions with mild and humid climates since the viability of its oocysts facilitates transmission and increases the infection rate of the parasite in these areas, often leading to high within-country variations in seroprevalence [4]. However, the limited availability of additional data, which is also a limitation of our research, leads to the retention of this assessment as a hypothesis. The availability of an expanded dataset, characterised by comprehensive annotations, would have facilitated a more precise evaluation of the risk factors underlying the parasite’s transmission routes in these patients.

## 5. Conclusions

There is a need for seroprevalence studies on *T. gondii* infection among HIV+ patients in Europe, including Greece. *T. gondii* infects approximately 30% of the world’s population, with a pooled prevalence of 35.8% among HIV+ patients. Here, we found a 31.61% positivity rate and significant correlations between *T. gondii* IgG presence and demographic factors such as gender and nationality among newly diagnosed HIV+ patients in Greece. Female HIV+ individuals and foreign or non-Greek HIV+ patients showed a higher likelihood of testing positive for *T. gondii* IgG. This could be due to varying hormonal influences between genders and exposure to environmental factors prior to their arrival in Greece, respectively. Comparative evaluation studies are crucial in identifying the most reliable diagnostic methods for *T. gondii* infection, leading to timely and appropriate patient care. The occurrence of false positives in ELISA and/or IFAT methods emphasises the need for such studies, and confirmatory testing using techniques like the LDBIO TOXO II Western Blot, which is noted for its high specificity and sensitivity. Latent *T. gondii* infection can reactivate and cause severe conditions like cerebral toxoplasmosis; therefore, early testing for *T. gondii* antibodies is crucial. Our study revealed a high seroprevalence of *T. gondii* among newly diagnosed HIV+ patients in Greece, underscoring the significance of incorporating toxoplasmosis in the screening guidelines of HIV+ patients by the Hellenic National Public Health Organization (NPHO). A positive result necessitates anti-toxoplasmosis prophylaxis alongside HAART, while a negative result emphasises the need for preventive measures against primary infection.

## Figures and Tables

**Table 1:** Relative sensitivity, specificity, Positive Predictive Value (PPV), Negative Predictive Value (NPV), precision, and accuracy values of the examined Enzyme-linked Immunosorbent Assay (ELISA) and Immunofluorescence Antibody Test (IFAT) immunoassays against the Western Blot (WB) method used as reference here, accompanied by their 95% confidence intervals in parentheses.

	Sensitivity	Specificity	PPV	NPV	Precision	Accuracy
**ELISA**	89.58%	69.23%	60.56%	92.65%	60.56%	76.26%
	(77.83–95.47%)	(59.13–77.77%)	(48.94–71.11%)	(83.91–96.82%)	(48.94–71.11%)	(68.54–82.57%)
**IFAT**	95.92%	88.68%	79.66%	97.92%	79.66%	90.97%
	(86.29–99.27%)	(81.25–93.40%)	(67.73–87.96%)	(92.72–99.63%)	(67.73–87.96%)	(85.41–94.54%)

**Table 2:** Statistical analysis summary of the demographic and immunological data sourced from the Human Immunodeficiency Virus positive (HIV+) patients of this study. The number of HIV+ patients with the given characteristics is reported accordingly, alongside the calculated relative risk and statistical significance followed by the respective *p*-values in parentheses. *** *p* < 0.001.

	HIV+ Patients Positive for *T. gondii* IgG (*n*=)	HIV+ Patients Negative for *T. gondii* IgG (*n*=)	Relative Risk	Statistical Significance (*p*)
**Gender**	22	20	2.24	*** (0.0009)
	(Female)	(Female)		
	26	85		
	(Male)	(Male)		
**Nationality**	29	33	2.34	*** (0.0006)
	(non-Greek)	(non-Greek)		
	18	72		
	(Greek)	(Greek)		
**Intravenous drug use**	6	15	0.89	− (0.8069)
	(With this	(With this		
	characteristic)	characteristic)		
	43	91		
	(Without this characteristic)	(Without this characteristic)		

## Data Availability

The original immunological data presented in the study are included in the Supplementary Material. Patient demographics are unavailable due to ethical restrictions.

## Supplementary Materials

The following supporting information can be downloaded at: https://www.mdpi.com/article/10.3390/pathogens13050375/s1, Supplementary File S1.
